# mtDNA-STING Axis Mediates Microglial Polarization *via* IRF3/NF-κB Signaling After Ischemic Stroke

**DOI:** 10.3389/fimmu.2022.860977

**Published:** 2022-04-05

**Authors:** Lingqi Kong, Wenyu Li, E Chang, Wuxuan Wang, Nan Shen, Xiang Xu, Xinyue Wang, Yan Zhang, Wen Sun, Wei Hu, Pengfei Xu, Xinfeng Liu

**Affiliations:** ^1^ Stroke Center and Department of Neurology, The First Affiliated Hospital of USTC, Division of Life Sciences and Medicine, University of Science and Technology of China, Hefei, China; ^2^ The First Affiliated Hospital of USTC, Division of Life Sciences and Medicine, University of Science and Technology of China, Hefei, China

**Keywords:** ischemia/reperfusion (I/R) injury, STING, microglia, polarization, neuroinflammation

## Abstract

Neuroinflammation is initiated in response to ischemic stroke, and is usually characterized by microglial activation and polarization. Stimulator of interferon genes (STING) has been shown to play a critical role in anti-tumor immunity and inflammatory diseases. Nevertheless, the effect and underlying mechanisms of STING on microglial polarization after ischemic stroke remain unclarified. In this study, acute ischemic stroke was simulated using a model of middle cerebral artery occlusion (MCAO) at adult male C57BL/6 mice *in vivo* and the BV2 microglia oxygen-glucose deprivation/reperfusion (OGD/R) model *in vitro*. The specific STING inhibitor C-176 was administered intraperitoneally at 30min after MCAO. We found that the expression of microglial STING was increased following MCAO and OGD/R. Pharmacologic inhibition of STING with C-176 reduced the ischemia/reperfusion (I/R)-induced brain infarction, edema and neuronal injury. Moreover, blockade of STING improved neurological performance and cognitive function and attenuated neuronal degeneration in the hippocampus after MCAO. Mechanistically, both *in vivo* and *in vitro*, we delineated that STING could promote the polarization of microglia towards the M1 phenotype and restrain M2 microglia polarization *via* downstream pathways, including interferon regulatory factor 3 (IRF3) and nuclear factor-κB (NF-κB). In addition, mitochondrial DNA (mtDNA), which is released to microglial cytoplasm induced by I/R injury, could facilitate microglia towards M1 modality through STING signaling pathway. Treatment with C-176 abolished the detrimental effects of mtDNA on stroke outcomes. Taken together, these findings suggest that STING, activated by mtDNA, could polarize microglia to the M1 phenotype following MCAO. Inhibition of STING may serve as a potential therapeutic strategy to mitigate neuroinflammation after ischemic stroke.

## Introduction

Ischemic stroke caused a huge social burden to the world with a high morbidity, disability rate and poor prognosis (1, 2). There is accumulating evidence indicating that post-ischemic inflammation is an essential process in the pathophysiology of ischemic stroke and closely related to the prognosis (3, 4). Therefore, it is important to gain a deeper understanding of neuroinflammation following ischemic stroke.

Microglia, known as the resident immune cell of the central nervous system (CNS), has emerged as a key mediator of neuroinflammation in the setting of ischemic stroke (5, 6). After stimulation, microglia could be polarized into the M1 or M2 form, which plays a dual role in brain injury, repair, and regeneration after ischemic stroke (7). M1 microglia secretes proinflammatory cytokines and chemokines, which can aggravate immune response and brain injury. In contrast, M2 microglia promotes the repair of the damaged tissue by producing anti-inflammatory cytokines (7, 8). Regulating the polarization of microglia and exploring the underlying mechanisms might provide novel insight into a theoretical basis for the study of ischemic stroke.

Stimulator of interferon genes (STING) is an endoplasmic reticulum adaptor protein that facilitates the transcription of numerous host defense genes, including type-I interferons (IFNs) and pro-inflammatory cytokines (9–11). STING can be directly activated by the second messenger cyclic guanosine monophosphate-adenosine monophosphate (cGAMP), which is produced from the cyclic GMP-AMP synthase (cGAS). cGAS, a central cellular cytosolic DNA sensor, can detect and bind to mitochondrial DNA (mtDNA) and nuclear DNA (nDNA) (9–11). STING is also known to bind double-stranded DNA (dsDNA) directly and subsequently instigate primary innate immune genes expression (12). Numerous studies have indicated that STING signaling pathway is involved in innate immune response, anti-tumor immunity and inflammatory diseases (13, 14). Intriguingly, STING has been reported to be expressed in microglia (15, 16). HDAC3-cGAS-STING signaling pathway was activated following ischemic stroke, which promoted the formation of a pro-inflammation microenvironment (17). Damage-associated molecular patterns (DAMPs) activated the microglial cGAS-STING signaling pathway and promoted microglia M1 polarization, while knockdown of cGAS could inhibit these effects (18). Moreover, nuclear dsDNA could drive cGAS signaling and further promote microglial inflammasome activation and pyroptosis to amplify the inflammation during cerebral ischemia (19). Cerebrovascular complications of tPA were aggravated *via* cGAS-STING activation and type I interferon response in ischemic brain (20). However, in these studies, cGAS was more concerned and explored in ischemic stroke, but there is no interfering targeting STING was performed. Whether STING modulates ischemic/reperfusion (I/R)-induced microglia polarization remains to be elucidated.

The mtDNA leakage following tissue injury has been reported to activate STING signaling (9, 21). mtDNA could be released to cytoplasm during the cerebral I/R process and act as a potent DAMPs (22, 23). However, to date, whether mtDNA activates STING after I/R injury has not been clarified. In this study, using the mice middle cerebral artery occlusion (MCAO) model and microglia oxygen-glucose deprivation/reperfusion (OGD/R) model, we examined whether STING could orchestrate neuroinflammation through transforming microglial polarization and explored the underlying mechanisms of STING activation.

## Methods and Materials

### Animals

Adult male C57BL/6 mice weighting 20-25g (6-8 weeks old) were purchased from Beijing Vital River Laboratory Animal Technology. All mice were housed in an environment with controlled light (12 h light/dark), temperature (temperature 23 ± 1°C) and humidity (humidity 55-60%) and provided ad libitum access to food and water. All experimental protocols were approved by the Animal Ethics Review Committee of The First Affiliated Hospital of the University of Science and Technology of China. The study was implemented according to the National Institute of Health Guide for the Care and Use of Laboratory Animals (NIH Publications No. 80-23, revised 1996). All mice were acclimated 1 week to the environment before use and randomized into groups. The experiment scheme is shown in **Supplementary Figure S1**. A total of 466 male mice were used in this study. The animal usage and the excluded reason are shown in **Supplementary Table S1**. At least 4 mice were analyzed for each data point.

### Transient Middle Cerebral Artery Occlusion Surgery

The middle cerebral artery occlusion (MCAO) model was induced by occluding the MCA using the intraluminal filament method (24). In brief, mice were anesthetized with 2% isoflurane in O_2_ (RWD Life Science, China). The right common carotid artery (CCA), internal carotid artery (ICA), and external carotid artery (ECA) were isolated through a midline cervical incision. The ECA was ligated, and a nylon suture with silicon (Beijing Cinontech Co., Ltd., China) was inserted through the ECA stump and next advanced into the ICA to occlude the origin of the middle cerebral artery (MCA). Mice underwent reperfusion after 90 min of occlusion. The body temperature was maintained at 37 ± 0.5°C by a homoeothermic blanket. To confirm the occlusion of MCA, mice were monitored for cerebral blood flow (CBF) with Laser Speckle Doppler Flowmetry (PeriCam PSI Z; Perimed, Sweden) contrast Imaging before, during, and after surgery (**Supplementary Figure S2**). A decline in regional CBF ≥ 75% of baseline was considered as a successful occlusion. Sham-operated mice were anesthetized and received the same surgical procedures except that the MCA was not occluded.

### Cell Culture and OGD/R Model

The mouse BV2 microglial cells and mouse HT22 hippocampal neurons were purchased from the Procell (Wuhan, China). Cells were grown in DMEM (Gibco, USA) medium supplemented with 10% FBS (Gibco, USA) and 1% penicillin-streptomycin (Gibco, USA) solution in a 37°C, 5% CO_2_ incubator. OGD/R was implemented according to our previous methods (24, 25). Briefly, cells were transferred to glucose-free and serum-free DMEM and then incubated in an anaerobic chamber equipped with Anaero Pack-Anaero (MITSUBISHI GAS CHEMICAL CO., INC. Japan). After cells were subjected to OGD for 2 h in the chamber, the medium was replaced by DMEM and returned to normal incubator for reoxygenation. Control microglia cells were cultured with normal oxygen-conditioned incubator for the same periods. For microglia-neuron cocultures, Transwell^®^plates (0.4-μm pore size, Corning, MA, USA) were used. HT22 cells were seeded in the lower chamber of the Transwell plates and cultured together with microglia, which were pretreated and underwent OGD/R.

### Drug Administration

STING inhibitor C-176 (6.1, 12.2, 24.4ug/g, MedChemExpress, USA) or vehicle (1% DMSO+corn oil) were administered intraperitoneally at 30min after MCAO surgery (16, 26). The mtDNA (5mg/kg), extracted from liver tissue by Mitochondrial DNA Isolation Kit (ab65321, Abcam, USA), was intracerebroventricular injected (right lateral ventricle, bregma: 0.23mm posterior, 1mm lateral, and 2.25 mm deep) immediately after MCAO modeling (27). To inhibit STING, BV2 cells were treated with 1 µM C-176 (28). Cells were collected 6 h after mtDNA (100 ng/ml) stimulation and used for subsequent analyses (29). The same volume of PBS was used as the control treatment.

### Small Interfering RNA (siRNA) Intracerebroventricular Injection and Cell Transfection

STING small interfering RNA (si-STING, sequence: 5’-CGAAAUAACUGCCGCCUCATT-3’) was used in our experiments according to a previous study (17). The 2′OMe+5′Chol+5′Cy5 modified STING siRNA (si-STING) and scrambled siRNA (si-NC) were synthesized by RiboBio (Guangzhou, China) for STING knockdown. After the mice were anesthetized with isoflurane, they were fixed with a stereotaxic apparatus (RWD Life Science, China). A 10µl stainless-steel microsyringe (Shanghai Gaoge Industry & Trade Co., Ltd., China) with siRNAs against STING (si-STING or si-NC, 1.5nmol in 3μl) was inserted into the right lateral ventricle (bregma: 0.23mm posterior, 1mm lateral, and 2.25 mm deep) at a flow rate of 300 nl/min. The injector was administered over 10 min and withdrawn slowly after the infusion, and MCAO surgical procedures were performed 48h after the infusion. After completion of the injection, the burr hole was sealed with bone wax and the incision was closed with sutures. For *in vitro* experiments, 1.25µl siRNA (20µM) and 3µl riboFECT™ CP Reagent (RiboBio, China) were mixed in 30µl riboFECT™ CP Buffer to generate a transfection mixture with a final concentration of 50nM, according to the manufacturer’s instruction. Then the 50nM transfection mixture of scrambled siRNA (si-NC) or interfering RNA (si-STING) was added into DMEM and cultured with BV2 microglia for 48 h. Immunofluorescent staining and western blot analysis were performed to examine the transfection efficiency.

### Neurobehavioral Test

Modified Neurological Severity Scores (mNSS) and Corner-turning test were performed to assess sensorimotor deficits and motor coordination at day 1, 3, 7, 14, and 21 after cerebral ischemia, as previously described (30). Behavioral function assessments were performed by two researchers who were blinded to the experiments.

Spatial learning and memory was investigated with the Morris Water Maze (MWM) test on days 22-28 after reperfusion (24, 31). Briefly, blind tests were performed on day 22 to exclude blind mice, in which mice were allowed to reach the platform with the flag when the platform was over the water. In the next 5 days, animals were trained to find the platform below the water in four trials. Five days later, the platform was removed and each mouse was explored to search the platform for 90s. The time spent in the target quadrant, the number of platform crossings, the escape latency to find the platform and the swim path length were recorded by the Smart video software (Panlab Harvard Apparatus, USA). The observer and recorder were blinded to animal grouping.

### Infarct Volume Evaluation and Brain Water Content

At 24 h after reperfusion, mice were anesthetized, and brains were quickly removed. The brains were frozen rapidly at -80°C with PBS for 6-8min, and coronally sliced in a brain mold and incubated in 2% 2,3,5-triphenyl tetrazolium chloride (TTC, Sigma Aldrich Inc., USA) solution for 10 min at 37°C (25). Then, the brain sections were photographed, and the infarct volume of each slice was determined using Image J software (NIH, USA) by an investigator blinded to the experimental design. To exclude the effects of brain edema, each slice infarct area was calculated as the volume of the contralateral hemisphere minus the non-infarcted volume of the ipsilateral hemisphere and summed to determine the whole infarct volume.

The brain water content was measured with the wet-dry method to evaluate the severity of brain edema at 24 h post-modeling (32). Brains were collected and quickly divided into the left hemisphere and the right hemisphere. Each hemisphere was weighed to get the wet weight and then weighed again at 105°C overnight to get the dry weight. The percentage of water content was calculated according to the following formula: [(wet weight-dry weight)/wet weight] ×100%.

### Immunofluorescence

The mice were deeply anesthetized and intracardially perfused with cold PBS and 4% paraformaldehyde (PFA). The brains were removed and fixed in 4% PFA at 4°C for 24 h, and then transferred into 30% sucrose solution until dehydration. Brains were embedded and frozen in the Tissue-Tek O.C.T. compound (Sakura Finetek, USA). The brains were cut into 15 μm or 20 μm frozen coronal sections with a Leica CM1950 cryostat for immunofluorescence staining. Sections were fixed with 4% PFA for 10 min, and then incubated with 10% donkey serum, 1% bovine serum albumin (BSA) and 0.1% Triton X-100 at room temperature for 1 h. Then the slices were incubated overnight at 4°C with indicated primary antibodies (**Supplementary Table S2**). After three washes with PBS, the slices were incubated for 2 h at room temperature with secondary antibodies (**Supplementary Table S2**). Coverslips were immunostained as brain slices did. Immunofluorescence images were acquired with an Olympus FV3000 fluorescence microscope (Japan). Quantification analysis of positive signals was performed with Image J software (NIH, USA) by an investigator blinded to the experimental design.

### TUNEL and Cells Live/Dead Staining

For NeuN and TUNEL co-staining, the frozen sections were first stained with NeuN antibody overnight at 4°C, followed by terminal deoxynucleotidyl transferase-mediated dUTP nick end-labeling (TUNEL) staining with One Step TUNEL Assay Kit (Beyotime, China), according to the manufacturer’s protocol. Three brain sections were examined per mouse, with each section containing three microscopic fields from the ischemic boundary zone. The means numbers of target cells were measured in sections per brain, by observers blinded to the experiments. Data were presented with the number of double-positive for TUNEL and NeuN neurons in the fields as cells/mm^2^. Meanwhile, HT22 cells death was determined with LIVE/DEAD™ Viability/Cytotoxicity Kit (Thermo Fisher Scientific, USA) after microglia-neuron co-cultures. The percentage of live cells (green) compared with the dead cells (red) was used to assess cell death. Images were captured using a confocal laser scanning microscope (Olympus FV3000, Japan).

### Fluoro-Jade C (FJC) Staining

Fluoro-Jade C (FJC, Millipore, USA) was performed at day 1 and day 28 after MCAO induction for identifying degenerated neurons (32, 33). Briefly, brain frozen sections were sequentially immersed in 1% NaOH/80% ethanol solution, 70% ethanol, and 0.06% potassium permanganate solution. Then, the sections were incubated with 0.0001% solution of FJC. Three brain slices were examined per mouse, and the number of FJC-positive neurons was quantified in three different fields of view for each section. Data were presented as TUNEL staining did.

### Quantitative Real-Time PCR

Total RNA from cells or tissue was extracted with TRIzol reagent (Invitrogen, USA) according to the manufacturer’s instruction and was reverse-transcribed into cDNA using HiScript III RT SuperMix for qPCR Kit (Vazyme, China). Then quantitative real-time PCR was performed using quantitative PCR (Light Cycler 96 System, Roche, China) with corresponding primers (**Supplementary Table S3**) in the presence of a fluorescent dye (AceQ qPCR SYBR Green Master Mix). The levels of mRNA were normalized in relevance to GAPDH.

### Western Blot Analysis

Total lysates of peri-infarct tissue or BV2 microglia were prepared using RIPA lysis buffer (Beyotime, China). Protein concentration was quantified by NanoPhotometer-N50 (IMPLEN GMBH, Germany). Equal amounts of protein were loaded and separated by 8-12% SDS-PAGE gel, then electrophoresed and transferred onto PVDF membranes (Millipore, USA). PVDF membranes were blocked with 5% BSA and incubated with primary antibodies overnight at 4°C and then incubated with appropriate HRP-conjugated secondary antibodies at room temperature for 1 h. The used antibodies were listed in **Supplementary Table S2**. Membranes were then incubated with ECL reagents (Willget biotech, China) before visualization using Azure 500 (Azure Biosystems, USA). β-Tubulin was served as the internal control. The immunoblots were analyzed by Image J software (NIH, USA).

### Statistical Analysis

All statistical tests were performed using SPSS 22.0 software (IBM, Armonk, NY, USA). Differences between groups were evaluated by the one-way ANOVA followed by Tukey’s *post hoc* test. The escape latency and swimming path length in MWM tests were analyzed using two-way ANOVA followed by Tukey’s *post hoc* test. All data were presented as mean ± SD. All tests were considered statistically significant at *P* < 0.05.

## Results

### STING in Microglia Was Upregulated With Stroke

**Figure 1:**
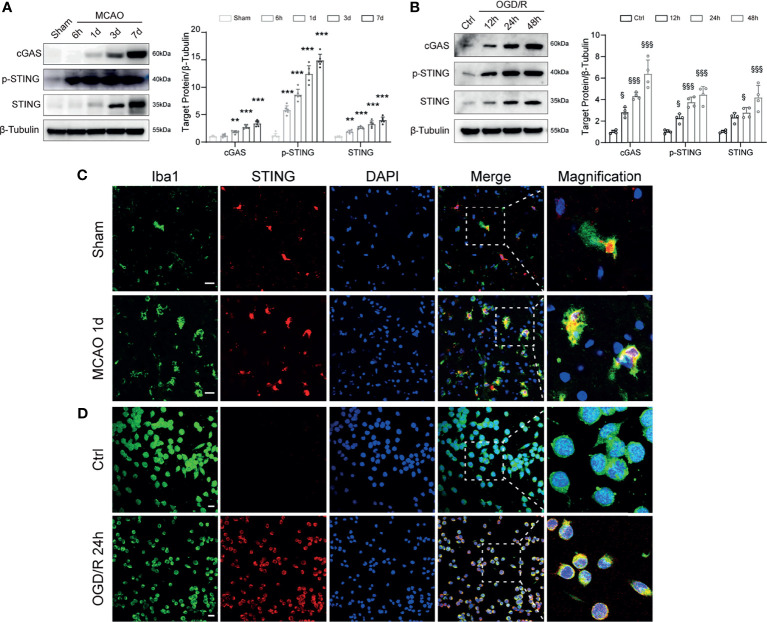
Temporal expression and cellular localization of STING following ischemic stroke. **(A)** Western blot and quantitative analysis of cGAS, p-STING and STING at 6 h, 1d, 3d, and 7d after MCAO. n = 6. **(B)** Western blotting showing cGAS, p-STING and STING expression at 12 h, 24 h, 48h after reoxygenation. n = 4. **(C, D)** STING/Iba1 double immunostaining in Sham and MCAO mice 1d after reperfusion and in Control and OGD/R BV2 microglia 24h after reoxygenation. STING signal was increased after I/R injury both *in vivo* and *in vitro*. n = 4. Data are expressed as mean ± SD. ^**^ *P* < 0.01, ^***^ *P* < 0.001 vs Sham group. ^§^ *P* < 0.05, ^§§§^ *P* < 0.001 vs Ctrl group. Scale bar = 20 μm.

We first investigated the expression change of STING in MCAO mice. Western blot results showed that the protein levels of cGAS, p-STING, and STING were significantly increased with time following MCAO (**Figure 1A**). BV2 microglia OGD/R model was used to mimic the *in vitro* conditions of I/R injury. Immunoblots showed that the expression levels of cGAS, p-STING, and STING in BV2 cells were significantly increased after OGD/R, and continuously increased as the reperfusion time prolongs (**Figure 1B**). Double immunostaining of STING with cell markers (Iba1, GFAP, or NeuN) demonstrated that STING was mainly expressed in microglia cells, rather than neurons or astrocytes and was increased at 1d after MCAO (**Figure 1C** and **Supplementary Figure S3**). Furthermore, STING was co-stained with Iba1 in BV2 cells. STING immunofluorescence signal was enhanced at 24h after reoxygenation (**Figure 1D**). Thus, our data indicated that STING was activated after I/R injury.

### Inhibition of STING Attenuated Brain Infarction and Neurological Deficits Following Ischemic Stroke

**Figure 2:**
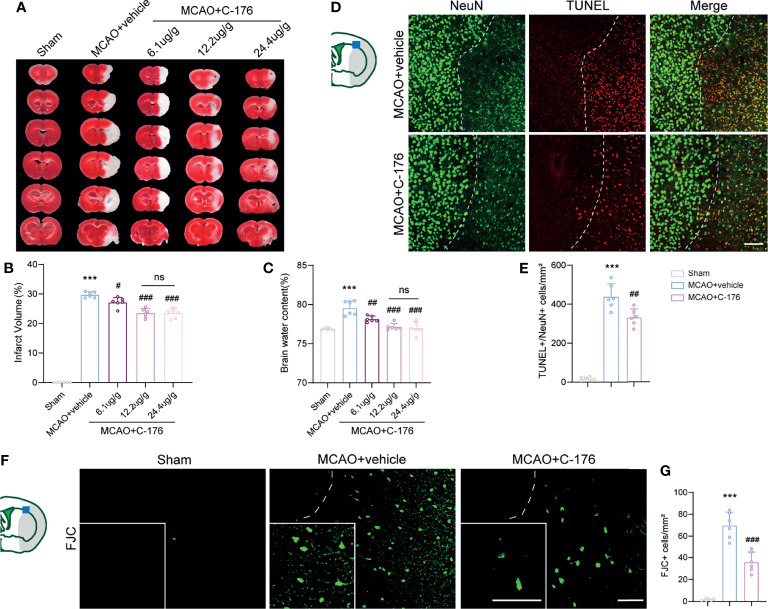
C-176 treatment rescued infarct volume, brain edema, and neuronal injury. **(A)** Representative images for TTC staining in the indicated groups.**(B, C)** Quantitative analysis of infarct volume and brain edema in treated mice 1d post-modeling. **(D, E)** Representative microphotographs and quantitative analysis of NeuN/TUNEL co-staining. The dotted lines designate infarct borderlines. **(F, G)** FJC staining showed that the density of FJC-positive cells was increased in the peri-infarct area, while C-176 treatment reversed this trend. Insets show a higher magnification view. n = 6. Data are expressed as mean ± SD. ^***^ *P* < 0.001 vs Sham group; ^#^ *P* < 0.05, ^##^ *P* < 0.01, ^###^ *P* < 0.001 vs MCAO+vehicle group. n. s., no significant difference. Scale bar = 50 μm.

To investigate the function of STING in the pathophysiological process of ischemic stroke, we intraperitoneally injected mice with C-176 to block STING. TTC staining showed that C-176 administration notably decreased brain infarction and brain edema in a dose-dependent manner 1d post-MCAO (**Figures 2A–C**, for brain infarction: *P* = 0.0344, *P* < 0.001 and *P* < 0.001, for brain edema: *P* = 0.0044, *P* < 0.001 and *P* < 0.001). The dosage of 12.2 and 24.4 ug/g were more effective than the dosage of 6.1 ug/g on brain infarction and cerebral edema 1d post-MCAO (**Figures 2A–C**). However, there were no significant differences between 12.2 ug/g and 24.4 ug/g dose on stroke outcomes (**Figures 2A–C**). Therefore, 12.2 ug/g dose was chosen for subsequent experiments.

We next investigated whether blockade of STING could rescue I/R-induced neuronal injury. TUNEL/NeuN staining was performed to assess neuronal apoptosis at 1d after reperfusion and found a significant decrease (~25%) in the number of apoptotic neurons in the MCAO+C-176 mice compared to the MCAO+vehicle mice (**Figures 2D, E**, *P* = 0.0023). We evaluated neuronal degeneration by FJC staining and found a decrease of ~49% in the number of neuronal degeneration in MCAO+C-176 mice at 1d post-modeling compared with that in MCAO mice (**Figures 2F, G**, *P* < 0.001).

### Blockade of STING Improved Long-Term Neurobehavioral Function

**Figure 3:**
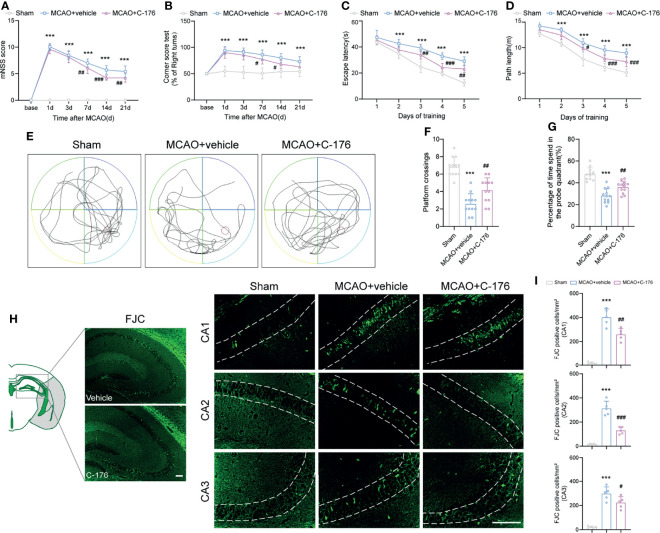
Inhibition of STING improved neurological performance and cognitive function after MCAO. **(A, B)** The mNSS and Corner test were assessed at day 1, 3, 7, 14, and 21 after MCAO. Mice were tested before MCAO surgery. MCAO 1-7 d: n = 15; MCAO 14-21 d: n = 10. Long-term cognitive functions were assessed by the Morris water maze. **(C, D)** The escape latency and swim path length were recorded at days 22-28 after MCAO. n = 12. **(E)** Representative swimming trajectories of the three groups in probe trials. **(F, G)** The crossovers of the platform location and the percentage of time spent in the probe quadrant. n = 12. **(H, I)** FJC staining was performed to assess the neuronal degeneration in hippocampus at day 28 after MCAO. n = 5. Data are expressed as mean ± SD. ^***^ *P* < 0.001 vs Sham group; ^#^ *P* < 0.05, ^##^ *P* < 0.01, ^###^ *P* < 0.001 vs MCAO+vehicle group. Scale bar = 100 μm.

The mNSS score was prominently improved in C-176-administered MCAO mice compared to the vehicle-treated MCAO mice on day 7, 14, and 21 post-stroke (**Figure 3A**, *P* = 0.0035, *P* < 0.001 and *P* = 0.0047). Similarly, the C-176-treated group exhibited a significant improvement in sensorimotor function with Corner test compared to the vehicle-treated group at 7 and 14 days after MCAO (**Figure 3B**, *P* = 0.0412, *P* = 0.0156). Cognitive function was assessed by the MWM test on days 22-28 after reperfusion. As shown in **Figures 3C, D**, the escape latency and the path length for the mice to find the hidden platform in the MCAO group were inferior to that of Sham-operated mice, suggesting that I/R injury induced severe learning and memory impairments. C-176-treated MCAO mice displayed a better performance with decreased escape latency and shorter path length on days 3 to 5 (for escape latency: *P* = 0.008, *P* < 0.001 and *P* = 0.0012, for path length: *P* = 0.0138, *P* < 0.001 and *P* < 0.001). Moreover, in the probe phase, treatment with C-176 conspicuously increased the crossovers of the platform and the time spent in the target quadrant compared to the MCAO+vehicle group (**Figures 3E–G**, *P* = 0.0096 and *P* = 0.0073).

We then used FJC staining to test whether STING could affect neuronal degeneration for a long time after ischemic stroke. Robust FJC staining in CA1, CA2, and CA3 regions of the hippocampus were found at 28 d after MCAO. The density of FJC-positive cells was increased after MCAO, while C-176 treatment significantly decreased the number of FJC-positive neurons in CA1, CA2 and CA3 regions of the hippocampus (**Figures 3H, I**, *P* = 0.0018, *P* < 0.001 and *P* = 0.0468). Taken together, blockade of STING may have substantially advantageous effects on long-term behavioral outcomes following ischemic stroke.

### Suppression of STING Inhibited Microglial M1 Polarization

**Figure 4:**
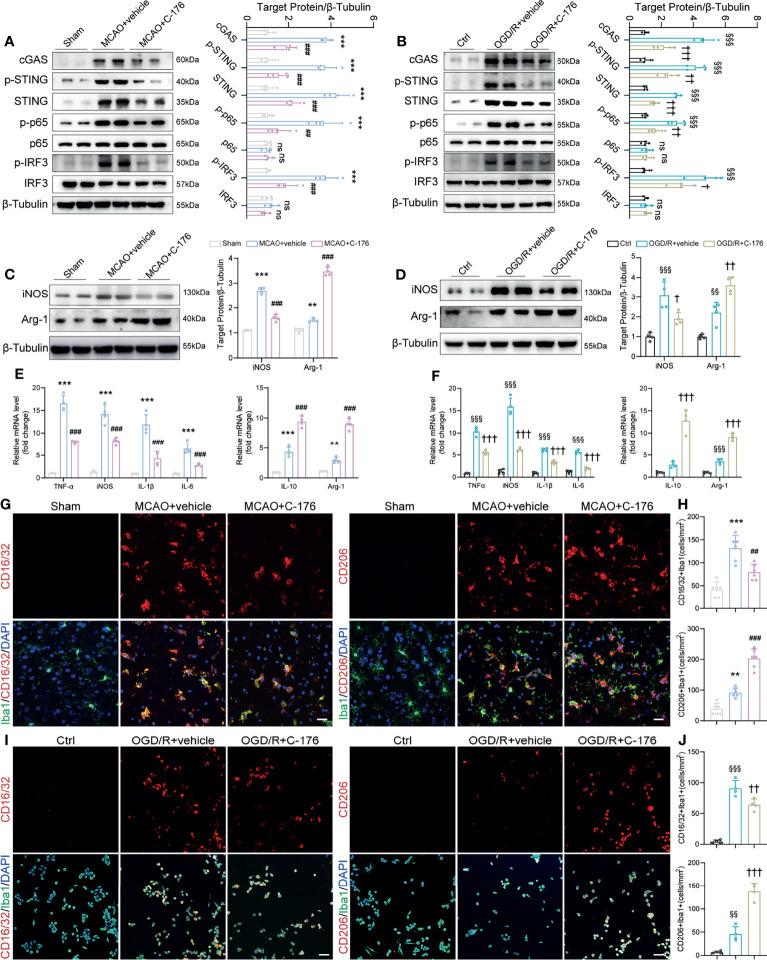
The STING inhibitor C-176 promoted M2 polarization after MCAO. **(A)** Immunoblotting analysis of cGAS, p-STING, STING, p-p65, p65, p-IRF3 and IRF3 in the peri-infract tissue of Sham, MCAO+vehicle and MCAO+C-176 mice 1d post-MCAO. n = 4. **(B)** Immunoblotting images and quantitative analysis of cGAS, p-STING, STING, p-p65, p65, p-IRF3 and IRF3 in BV2 microglia of Ctrl, OGD/R+vehicle and OGD/R+C-176 group 24h after reoxygenation. n = 4. **(C, D)** Western blot and quantification analysis for iNOS and Arginase-1 in treated mice 3d post-MCAO and BV2 microglia 24h post-reoxygenation. n = 4. **(E, F)** mRNA expression of pro-inflammatory genes (TNF-α, iNOS, IL-1β and IL-6) and anti-inflammatory genes (IL-10 and Arg-1) were measured by real-time PCR 3d after MCAO and 24h after OGD/R. n = 4. **(G)** Representative confocal images of two microglia phenotypes were obtained from the peri-infarct cortex. **(H)** Quantification of CD16/32 positive M1 microglia and CD206 positive M2 microglia in the treated mice. n = 6. **(I, J)** Representative immunostained images and statistical analysis of M1 state (CD16/32^+^/Iba1^+^) and M2 state (CD206^+^/Iba1^+^) BV2 microglia. n = 4. Data are expressed as mean ± SD. ^**^ *P* < 0.01, ^***^ *P* < 0.001 vs Sham group; ^##^ *P* < 0.01, ^###^ *P* < 0.001 vs MCAO+vehicle group. ^§§^ *P* < 0.01, ^§§§^ *P* < 0.001 vs Ctrl group; ^†^ *P* < 0.05, ^††^ *P* < 0.01, ^†††^ *P* < 0.001 vs OGD/R+vehicle group. n. s., no significant difference. Scale bar = 20 μm.

Having demonstrated that STING modulates functional outcomes after stroke, we sought to explore whether STING could affect post-ischemic inflammation through microglial polarization. We found that the protein levels of cGAS, p-STING, STING, p-p65, and p-IRF3 were increased substantially in MCAO mice and OGD/R microglia; whilst the STING-related proteins expression levels were remarkably decreased with C-176 administration (**Figures 4A, B**). Moreover, immunoblots showed that the levels of the M1-related marker iNOS decreased and the M2-related marker Arginase-1 increased with C-176 treatment 3d after MCAO and 24h after OGD/R (**Figure 4C**, both *P* < 0.001, **Figure 4D**, *P* = 0.0133 and *P* = 0.0018). Real-time PCR analysis indicated that the levels of M1 phenotype factors (TNF-α, iNOS, IL-1β, IL-6) and the M2 phenotype factors (IL-10 and Arg-1) increased at 3d after reperfusion (**Figure 4E**, *P* < 0.001, *P* < 0.001, *P* < 0.001, *P* < 0.001, *P* < 0.001 and *P* = 0.006). However, C-176 treatment notably mitigated the expression of M1 phenotype related genes and markedly enhanced the expression of M2 phenotype related genes (**Figure 4E**, all *P* < 0.001). Consistent with observation *in vivo*, C-176 incubation markedly upregulated the expression level of M2 phenotype factors and notably suppressed the levels of M1 phenotype factors in BV2 microglia after 24h reoxygenation (**Figure 4F**, all *P* < 0.001).

We then investigated the effects of STING on microglial phenotype transition. CD16/32 and CD206 staining were used to examine the polarization of microglia in the peri-infarct cortex. As depicted in **Figures 4G, H**, the number of CD16/32 and Iba1 double-positive M1-like cells was significantly increased on day 3 after MCAO when compared with the Sham group, which was markedly decreased in the MCAO+C-176 group (*P* = 0.0019). On the other hand, the number of CD206 and Iba1 double-positive M2-like cells was further increased by C-176 administration at 3 days after MCAO (**Figures 4G, H**, *P* < 0.001). Coincident with the observation on day 3 after MCAO, C-176 treatment also notably decreased the CD16/32-positive microglia and increased the CD206-positive microglia on day 7 after MCAO (**Supplementary Figure S4**, *P* = 0.0210 and *P* = 0.0007). We further performed immunostaining for the BV2 cells to evaluate the effects of C-176 on the M1/M2 polarization state at 24h after OGD/R. Blockade of STING reduced the number of CD16/32^+^ microglia cells, and increased the number of CD206^+^ microglia cells (**Figures 4I, J**, *P* = 0.0074 and *P* < 0.001). OGD/R induced a substantial increment of neuronal death as determined by quantification of live/dead staining, while incubation with C-176 maximally preserved neuronal viability (**Supplementary Figure S5**, *P* = 0.0026). These results demonstrated that C-176 alleviated cerebral I/R injury by promoting the microglial polarization to M2 state.

### Knockdown of STING in Microglia Attenuated I/R-Induced Neuroinflammation and Brain Injury

**Figure 5:**
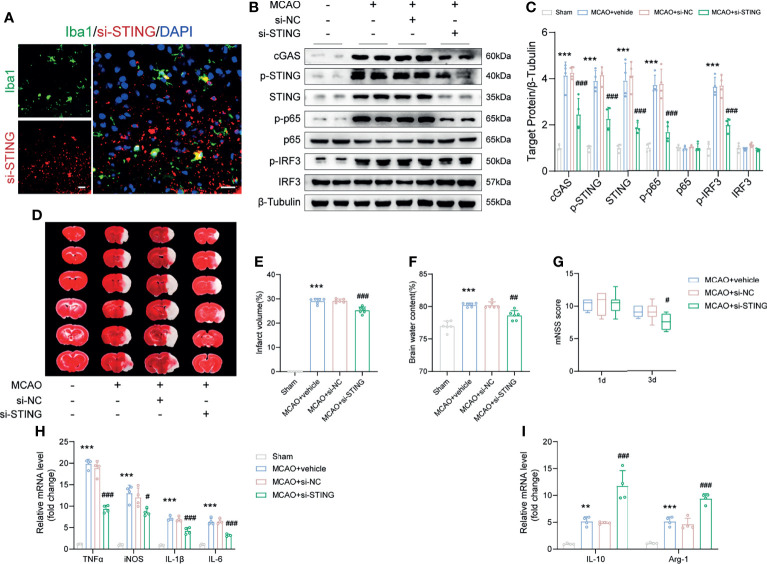
STING knockdown ameliorated brain infarction and edema, promoted neurological recovery and rescued the expression of anti-inflammatory genes. **(A)** si-STING (red) co-stained with Iba1 (green) 1d post-MCAO in mice receiving Cy5-conjugated si-STING. **(B, C)** Immunoblots and quantitative analysis of STING-related proteins, including cGAS, p-STING, STING, p-p65, p65, p-IRF3 and IRF3 of Sham, MCAO+vehicle, MCAO+si-NC and MCAO+si-STING mice 1d after MCAO. n = 4. **(D-F)** Representative images of TTC staining and quantitative analysis of infarct volume and brain edema 1d post-MCAO. n = 6. **(G)** mNSS score of the indicated groups at day 1 and day 3 after MCAO. n = 8. **(H, I)** Relative mRNA expression of M1 microglia-specific transcripts (TNFα, iNOS, IL-1β and IL-6) and M2 microglia-specific transcripts (IL-10 and Arg-1). n = 4. Data are expressed as mean ± SD. ^**^ *P* < 0.01, ^***^ *P* < 0.001 vs Sham group; ^#^ *P* < 0.05, ^##^ *P* < 0.01, ^###^ *P* < 0.001 vs MCAO+si-NC group. Scale bar = 20 μm.

To further verify the role of STING post-MCAO, siRNA was administrated i.c.v to silence STING. Double immunostaining images at the siRNA injection site and the surrounding area showed that Cy5-conjugated si-STING was co-stained with microglia (**Figure 5A**). And we found that the levels of p-STING and STING were suppressed by si-STING (**Figures 5B, C**, both *P* < 0.001). Similarly, si-STING transfection could effectively knockdown p-STING and STING expression in BV2 microglia 24h after OGD/R (**Supplementary Figure S6**, *P* = 0.0099 and *P* = 0.0087). These results suggested that si-STING could transfect into microglia and knockdown of STING expression.

Genetic knockdown of STING decreased the infarct volume and brain edema, and alleviated neurological deficits post-MCAO (**Figures 5D–G**, *P* < 0.001, *P* = 0.0011 and *P* = 0.0207). Moreover, administration with si-STING significantly suppressed the mRNA level of M1 phenotype markers (TNF-α, iNOS, IL-1β, and IL-6) 1d post-MCAO when compared to the MCAO+si-NC group (**Figure 5H**, *P* < 0.001, *P* = 0.0291, *P* < 0.001 and *P* < 0.001). We also found that silence STING upregulated the expression of M2 phenotype mRNA markers (IL-10 and Arg-1), while injection si-NC had no such effects (**Figure 5I**, both *P* < 0.001).

### Inhibition of STING Retarded the Expression of mtDNA After MCAO

**Figure 6:**
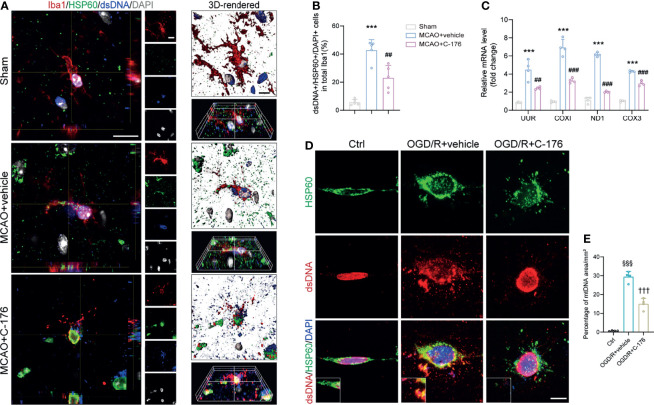
Blockade of STING suppressed microglial mtDNA leakage after MCAO. **(A, B)** Representative confocal, 3D-reconstructed images of dsDNA^+^/HSP60^+^/Iba1^+^ microglia and quantification of microglial mtDNA 1d post-MCAO. Images were reconstructed from confocal images using Imaris software. n = 5. **(C)** Relative mRNA expression of mtDNA-specific genes (UUR, COXI, ND1 and COX3) 1d after MCAO. n = 4. **(D, E)** BV2 microglia cells were stained with anti-dsDNA and anti-HSP60 to detect mtDNA at 24h after reoxygenation. Representative dsDNA/HSP60 double immunostaining images and quantification of mtDNA. n = 4. Data are expressed as means ± SD. ^***^ *P* < 0.001 vs Sham group; ^##^ *P* < 0.01, ^###^ *P* < 0.001 vs MCAO+vehicle. ^§§§^ *P* < 0.001 vs Ctrl group; ^†††^ *P* < 0.001 vs OGD/R+vehicle group. Scale bar = 10 μm.

Recently, it has been reported that mtDNA could release into the neuronal cytoplasm and participate in the pathogenesis of amyotrophic lateral sclerosis (ALS) (34). As a potent DAMPs, mtDNA is closely involved in the immune process after stroke (35). To this end, we triple-labeled stained against dsDNA, HSP60 (a mitochondrial marker), and Iba1 to assess whether mtDNA could leak into the microglial cytoplasm after I/R injury. Immunofluorescence and 3D-reconstructed images revealed that quantities of mtDNA were released into microglial cytoplasm following MCAO, which were alleviated by treatment with C-176 (**Figures 6A, B**, *P* = 0.0016). A similar trend was found in the cell experiments. Incubation with C-176 remarkably reduced the level of mtDNA, which was released into microglial cytoplasm induced by OGD/R ictus (**Figures 6D, E**, *P* < 0.001). I/R injury also induced the levels of mtDNA mRNA markers (UUR, COXI, ND1, and COX3) to noticeably increase, and these increments were all reversed by C-176 injection (**Figure 6C**, *P* = 0.0030, *P* < 0.001, *P* < 0.001 and *P* < 0.001).

### MCAO-Induced mtDNA Was Responsible for Microglial Polarization Through the STING Pathway

**Figure 7:**
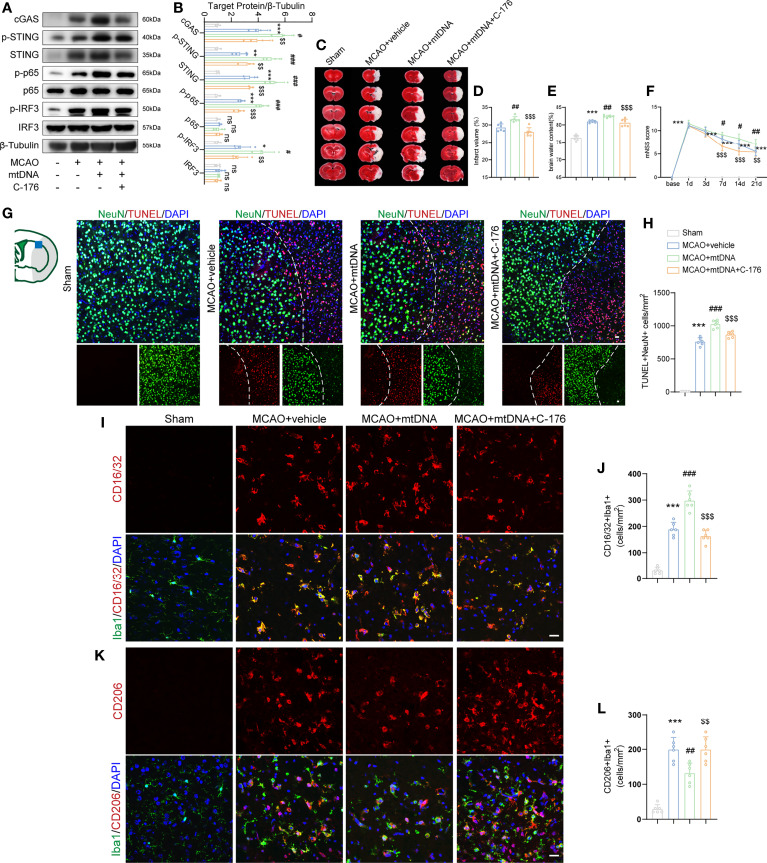
mtDNA induced microglial M1 polarization by activating STING signaling pathway. **(A, B)** Western blot and quantification analysis of cGAS, p-STING, STING, p-p65, p65, p-IRF3 and IRF3 in Sham-, MCAO+vehicle-, MCAO+mtDNA-, and MCAO+mtDNA+C-176-treated mice 1d after reperfusion. n = 4. **(C-E)** Representative TTC staining images and quantification of infarction volume and brain edema 1d post-MCAO. n = 6. **(F)** mNSS score was assessed at day 1, 3, 7, 14 and 21 after MCAO. MCAO 1-7 d: n = 10; MCAO 14-21 d: n = 8. **(G, H)** TUNEL (red) and NeuN (green) double immunostaining with quantitative analysis. Dotted line designates the infarct borderline. n = 6. **(I–L)** Representative confocal images and quantification of M1 modality microglia (CD16/32^+^/Iba1^+^) and M2 modality microglia (CD206^+^/Iba1^+^) in the indicated groups. n = 6. Scale bar = 20 μm. Data are expressed as mean ± SD. ^*^ *P* < 0.05, ^**^ *P* < 0.01, ^***^ *P* < 0.001 vs Sham group; ^#^ *P* < 0.05, ^##^ *P* < 0.01, ^###^ *P* < 0.001 vs MCAO+vehicle group; ^$$^ *P* < 0.01, ^$$$^ *P* < 0.001 vs MCAO+mtDNA group. n. s., no significant difference.

mtDNA has been found to be capable of triggering STING signaling (21). We next sought to explore the role of microglial mtDNA in the STING-mediated I/R injury. Mice were received an intracerebroventricular injection of 5 mg/kg mtDNA. Compared with the vehicle-treated MCAO mice, mtDNA injection could markedly enhance the protein levels of cGAS, p-STING, STING, p-p65 and p-IRF3 1d after MCAO, which were alleviated by C-176 administration (**Figures 7A, B**). A similar tendency was observed in BV2 cells that C-176 incubation notably reversed the increments of STING-related proteins induced by mtDNA treatment (**Supplement Figure S7**). In addition, aggravated brain infarction and brain edema were observed as a result of mtDNA administration in MCAO mice, which was reversed by C-176 administration (**Figures 7C–E**, both *P* < 0.001). mtDNA injection remarkably worsened neurobehavioral performance in MCAO mice compared to the MCAO+vehicle group throughout the testing periods (**Figure 7F**, *P* = 0.0301, *P* = 0.0317 and *P* = 0.0067**)**. The mtDNA+C-176 mice displayed significantly better neurological performance than the mice in the mtDNA group on days 7 to 21 post-MCAO (**Figure 7F**, *P* < 0.001, *P* < 0.001 and *P* = 0.0036). As illustrated in **Figures 7G, H**, MCAO mice injected with mtDNA induced the increment of TUNEL-positive cells at 1d after reperfusion, which was alleviated by treatment of C-176 (*P* < 0.001).

We further evaluated the effects of STING activation, which is induced by mtDNA injection, on microglial polarization in ischemic stroke. Immunofluorescent staining results showed that mtDNA treatment increased the numbers of Iba1^+^CD16/32^+^ pro-inflammatory microglia and reduced Iba1^+^CD206^+^ anti-inflammatory microglia at 3d after MCAO compared with vehicle-treated mice (**Figures 7I–L**, *P* < 0.001 and *P* = 0.0040). However, co-administration of mtDNA and C-176 could suppress the number of the M1-specific marker CD16/32 and increase the number of the M2-specific marker CD206 in microglia after MCAO attack (**Figures 7I–L**, *P* < 0.001 and *P* = 0.0040). These results suggested that mtDNA could trigger microglial phenotype shift towards the M1 modality through activation of STING, resulting in neurological function deterioration following ischemic stroke.

## Discussion

**Figure 8:**
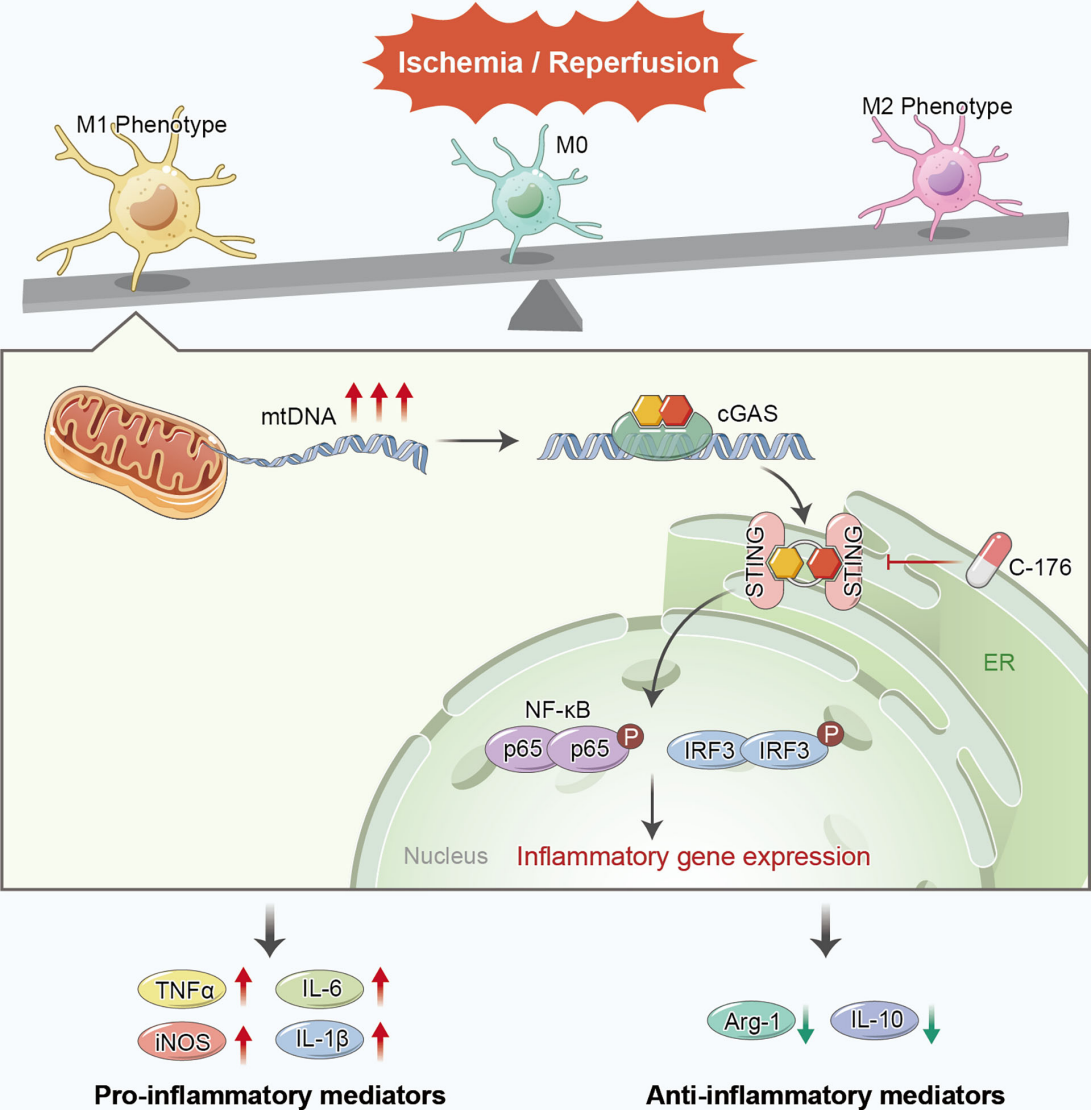
Proposed mechanism of STING-mediated microglial polarization following ischemic stroke. I/R injury induces the leakage of mtDNA to cytoplasm and the activation of STING in microglia. Blockade of STING shifts the microglia toward M2 modality through IFR3 and NF-κB, thereby rescuing neuroinflammation and stroke outcomes. mtDNA can promote microglial polarization to the M1 phenotype *via* activating STING signaling pathway.

In the current study, we found that STING was triggered and mainly located in microglia after ischemic stroke. Pharmacological blockade of STING with C-176 remarkably alleviated brain infarct volume, brain edema, neuronal apoptosis and degeneration, and thus recovered short-term and long-term neurological function. Moreover, inhibition of STING remarkably decreased the number of M1 phenotype microglia and facilitated microglial phenotype towards the M2 phenotype following cerebral I/R injury. In addition, we demonstrated that the microglial mtDNA, which escaped into the cytoplasm under I/R stimulation, could promote microglial polarization switch to M1 phenotype *via* STING signaling pathway. Blockade of STING remarkably shifted microglial polarization towards the M2 phenotype and alleviated neuroinflammation (**Figure 8**).

STING is a dimeric transmembrane protein at the endoplasmic reticulum (ER) with 42-kDa (14). Research on the immune role of the STING pathway in CNS disorders has grown in recent years (34, 36, 37). However, little is known about the role of STING in the pathological process of ischemic stroke. Here we showed that STING expression gradually increased at least 7 days following ischemic stroke. In agreement with our study, in the hypoxic-ischemic encephalopathy (HIE) model, M. Gamdzyk and colleagues demonstrated that the expression of STING was significantly increased from 6 h to 7d after HIE (38). Yet, a study examining the function of STING in subarachnoid hemorrhage (SAH) mice showed that the level of STING was increased post-SAH, peaked at 24h and gradually declined (16). Simultaneously, our immunofluorescence staining results confirmed that STING was mainly distributed in microglia after MCAO, which was similarly evaluated by the work of Peng and colleagues under the SAH condition (16). However, there was research showing that STING was located in neurons and astrocytes in HIE and traumatic brain injury (TBI) models (38, 39). The different models and complex microenvironments in the brain may be responsible for the different expression and location patterns of STING.

C-176, a highly potent and selective small molecule antagonist of STING, is widely used to block STING. C-176 could covalently target transmembrane cysteine residue 91 and thereby block the activation-induced palmitoylation of STING (26). It is reported that C-176 could effectively attenuate STING-associated autoinflammatory disease and early brain injury *via* intraperitoneally injection (16). So, intraperitoneally delivery of C-176 was applied to inhibit STING in our study. Pharmacological inhibition of STING with C-176 significantly alleviated neuroinflammation, thereby reducing brain water content and neurological deficits in SAH mice (16). Inhibition of STING with C-176 abrogated the increased level of proinflammatory cytokines/chemokines secretion in BMDMs in both young and aged mice (29). In this study, we found that pharmacological inhibition of STING with C-176 could suppress neuroinflammation and improve stroke outcomes. Li et al. showed that activation of cGAS-STING signaling could drive microglial inflammasome production and microglia pyroptosis to amplify the inflammation during cerebral I/R injury (19). However, the exact mechanisms of STING on neuroinflammation in ischemic stroke are not completely understood.

Neuroinflammatory, which responses to post-ischemia, is characterized by rapid activation of resident microglia cells (40). Microglia is the chief innate immune cell within the CNS and the most potent modulator of CNS repair and regeneration (5, 7, 41). But microglia cells play an apparent double-edged sword role in the CNS. Recent studies have reported that regulation of the balance between M1 and M2 phenotypes is a promising therapeutic strategy for brain ischemia (42–44). Considering that STING contributes to microglia/macrophages polariztion in SAH and colitis (16, 45), we speculated that STING might mediate microglial polarization in ischemic stroke. Indeed, our results showed that the gene and protein expressions of M1 phenotype in microglia was increased at 3 days post-MCAO, while the genes and proteins expression of M2 phenotype was suppressed. Additionally, inhibition of STING with C-176 notably promoted the M2 phenotype but reduced the M1 phenotype to exert an anti-inflammatory function. In this study, we chose the third-day time point to analyze microglial polarization states. The reasons are as follows: i) M1 microglia gradually increased over time from day 3 onward and remained elevated for at least 14 days after ischemia; ii) M2 microglia gradually increased beginning 1 to 3 days, peaked by 3 to 5 days and then decreased at 7 days after MCAO (7, 46).

Another major observation in this study was that STING could mediate microglial polarization through activating IRF3 and NF-κB pathways. The phosphorylated STING recruits and activates IRF3, ultimately, IRF3 enters the nucleus and exerts its function in the type-I interferon responses (10, 47). Meanwhile, STING could mediate the production of NF-κB-driven inflammatory genes and subsequently immune responses (48). It is reported that IRF3 and NF-κB could regulate microglial polarization after ischemic stroke (49).We speculated that STING might influence microglial polarization through activating IRF3 and NF-κB pathways following MCAO. Indeed, as depicted in western blot results, the protein expression of p-p65 and p-IRF3 was increased post-MCAO, and C-176 administration suppressed this increment. Furthermore, we noted that the downregulation of p-p65 and p-IRF3 was accompanied with decreasing microglial M1 phenotype polarization and growing M2 phenotype polarization. Intriguingly, it is reported that STING could recruit and improve NLRP3 localization in the endoplasmic reticulum, and remove NLRP3 polyubiquitination, thereby promoting the inflammasome activation (50). NLRP3 inflammasome has been proposed as a critical mediator of microglial M1/M2 polarization post-ischemic stroke (51). Therefore, NLRP3 inflammasome may be involved in STING-medicated microglial polarization. Accumulating evidences demonstrated that metabolic reprogramming and autophagy could orchestrate microglia activation and polarization (52). A recent investigation has declared that STING could orchestrate metabolic reprogramming of macrophages *via* HIF-1α during bacterial infection (53). Moreover, STING activation induces autophagy dysfunction in TBI and sepsis-related acute lung injury mice (39, 54). In view of these, STING might affect the polarization states of microglial cells through regulating other pathways, such as metabolic reprogramming and autophagy. Further researches are needed to elucidate the specific way of STING mediated microglial polarization.

Mitochondria dysfunction, including mtDNA damage, depletion and release, plays a critical role in ischemic cascades (55). mtDNA could activate the cGAS-STING immune pathway, subsequently regulate innate immune response and sterile inflammation (17, 21, 56). Here, we verified that the mtDNA could be released into the microglial cytoplasm and activated the STING signaling pathway during the cerebral I/R process. Previous studies have shown that STING was able to complex with self or pathogen-related signal-stranded DNA (ssDNA) and dsDNA, possibly by interacting with DNA through its cytoplasmic tail (12).However, as a dsDNA, whether mtDNA can directly bind to STING under ischemia conditions needs further study. Mitochondria are present in varieties of cells in CNS and comprise the intracellular cores for energetics and viability (57). Neurons and astrocytes can exchange damaged mitochondria with each other for disposal and recycling after stroke (58, 59). Furthermore, fragmented mitochondria released from microglia can trigger A1 astrocytic phenotype and propagate neuronal death (60). Thus, the elevated mtDNA in microglial cytoplasm of ishchemic brain may be partly from astrocytes and neurons through cell to cell conmmunication. Follow-up studies to elucidate the source of mtDNA in microglia cytoplasm may be envisioned.

## Conclusions

In summary, the present study demonstrated that STING could orchestrate neuroinflammation after ischemic stroke *via* mediating microglial polarization to M1 phenotype. Blockade of STING could inhibit inflammatory responses and provide a neuroprotective effect following ischemic stroke.These findings indicated that STING might be a therapeutic target for ischemic stroke.

## Data Availability Statement

The original contributions presented in the study are included in the article/**Supplementary Material**. Further inquiries can be directed to the corresponding authors.

## Ethics Statement

The animal study was reviewed and approved by The First Affiliated Hospital of the University of Science and Technology of China.

## Author Contributions

XL and PX designed the research. LK and PX drafted the original manuscript. LK and WL performed the animal experiments. EC and WW performed the cellular experiments. NS, XX, and XW contributed to western blots and immunostaining. WL and LK analyzed the data. WS and YZ gave critical comments for the research. WH, PX, and XL conceptualized and supervised the study. All authors read and approved the final manuscript.

## Funding

This work was supported by the National Natural Science Foundation of China (Nos. U20A20357 and 82101368), the Anhui Provincial Natural Science Foundation (Nos. 2008085QH368, 2108085MH271 and 2108085MH272), the Fundamental Research Fund for the Central Universities (No. WK9110000056), and the Program for Innovative Research Team of The First Affiliated Hospital of USTC.

## Conflict of Interest

The authors declare that the research was conducted in the absence of any commercial or financial relationships that could be construed as a potential conflict of interest.

## Publisher’s Note

All claims expressed in this article are solely those of the authors and do not necessarily represent those of their affiliated organizations, or those of the publisher, the editors and the reviewers. Any product that may be evaluated in this article, or claim that may be made by its manufacturer, is not guaranteed or endorsed by the publisher.

## Supplementary Material

Supplementary Figure 1 Experimental design and animal groups. i.p., intraperitoneal injection; i.c.v, intracerebroventricular injection; MCAO, middle cerebral artery occlusion; RT-PCR, real-time polymerase chain reaction; siRNA, short interfering RNA; STING, Stimulator of IFN genes; TTC, 2,3,5-triphenyl tetrazolium chloride solution; mtDNA, Mitochondrial DNA.

Supplementary Figure 2 Occlusion of MCA decreased cerebral blood flow (CBF). Representative images and quantitative analyses showed the CBF before ischemia, during ischemia, and 24 hours after reperfusion. Data are expressed as mean ± SD, n = 6. ^***^ *P* < 0.001 vs Baseline group. ^###^ *P* < 0.001 vs Ischemia group.

Supplementary Figure 3 Colocalization of STING with GFAP or NeuN in the peri-infarct cortex of mice. STING was undetectable in astrocytes and neurons. Scale bar = 20 μm.

Supplementary Figure 4 Effect of inhibit STING on microglia polarization at 7 days post-MCAO. Representative images of double immunostaining and quantitative analyses of microglia polarization. M1-phenotype: CD16/32^+^ (red) and Iba1^+^ (green); M2-phenotype: CD206^+^ (red) and Iba1^+^ (green). Data are expressed as mean ± SD, n = 6. ^**^ *P* < 0.01, ^***^ *P* < 0.001 vs Sham group; ^#^ *P* < 0.05, ^###^ *P* < 0.001 vs MCAO+vehicle group. Scale bar = 20 µm.

Supplementary Figure 5 Pharmacological inhibition of STING suppressed neuronal death *in vitro*. Neurons were co-cultured with Control microglia, OGD/R microglia, or OGD/R microglia treated with C-176 (1 μM) for 24 h. Neuronal viability of the three groups was measured by Live/Dead staining. Data are expressed as mean ± SD, n = 4. ^§§§^ *P* < 0.001 vs Ctrl group; ^††^ *P* < 0.01vs OGD/R+vehicle group. Scale bar = 200 µm.

Supplementary Figure 6 Transfection of siRNA restrained STING expression *in vitro*. **(A)** Colocalization of Cy5-conjugated si-STING with Iba1 24h after OGD. **(B)** Western blotting and quantitative analysis for cGAS, p-STING and STING. Data are expressed as mean ± SD, n = 4. ^§§§^ *P* < 0.001 vs Control group; ^†^ *P* < 0.05, ^††^ *P* < 0.001 vs OGD/R+si-NC group. Scale bar = 50 µm.

Supplementary Figure 7 mtDNA pre-treatment promoted STING expression *in vitro*. BV2 cells were pre-treated with mtDNA (100 ng/ml) for 6h before C-176 incubation. Immunoblots and densitometry analysis of cGAS, p-STING, STING, p-p65, p65, p-IRF3 and IRF3 in Ctrl, OGD/R+vehicle, OGD/R+mtDNA, OGD/R+mtDNA+C-176 groups. Data are expressed as mean ± SD, n = 4. ^§§§^ *P* < 0.001 vs Ctrl group; ^†^ *P* < 0.05, ^††^ *P* < 0.01, ^†††^ *P* < 0.001 vs OGD/R+vehicle group; ^$$^ *P* < 0.01, ^$$$^ *P* < 0.001 vs OGD/R+mtDNA group.

Supplementary Table 1 Animal usage and mortality of all the experimental groups.

Supplementary Table 2 Antibodies used in this study.

Supplementary Table 3 Sequences of the PCR primers used in the study.

## Abbreviations

ALS, Amyotrophic lateral sclerosis; ANOVA, One-way analysis of variance; Arg-1, Arginase-1; CBF, Cerebral blood flow; CCA, Common carotid artery; cGAMP, Cyclic guanosine monophosphate-adenosine monophosphate; cGAS, Cyclic GMP-AMP synthase; CNS, Central nervous system; DAPI, 4’,6-Diamidino-2-phenyl-indole; DMEM, Dulbecco’s modified Eagle’s medium; dsDNA, Double-stranded DNA; ECA, External carotid artery; FJC, Fluoro-Jade C; GAPDH, Glyceraldehyde-3-phosphate dehydrogenase; GFAP, Glial fibrillary acidic protein; HIE, Hypoxic-ischemic encephalopathy; I/R, Ischemia–reperfusion; Iba1, Ionized calcium-binding adaptor molecule 1; ICA, Internal carotid artery; i.c.v, Intracerebroventricular injection; iNOS, Inducible nitric oxide synthase; i.p., Intraperitoneal injection; IRF3, Transcription factor 3; MCAO, Middle cerebral artery occlusion; mNSS, Modified neurological severity score; mtDNA, Mitochondrial DNA; NeuN, Neuronal nuclei; NF-κB, Nuclear factor-κB; NIH, National Institute of Health; NLRP3, Nucleotide-binding oligomerization domain (Nod)-like receptor pyrin domain containing 3; OGD/R, Microglia oxygen-glucose deprivation/reperfusion; PBS, Phosphate-buffered saline; PFA, Paraformaldehyde; qPCR, Quantitative polymerase chain reaction; SAH, Subarachnoid hemorrhage; SD, Standard deviation; si-NC, Scrambled siRNA; siRNA, Short interfering RNA; ssDNA, Signal-stranded DNA; STING, Stimulator of IFN genes; TBI, Traumatic brain injury; TBST, Tris-buffered saline containing Tween-20; TTC, 2,3,5-Triphenyltetrazolium chloride; TUNEL, Terminal deoxynucleotidyl transferase-mediated dUTP nick end-labeling.

## Glossary

**Table:**

ALS	Amyotrophic lateral sclerosis
ANOVA	One-way analysis of variance
Arg-1	Arginase-1
CBF	Cerebral blood flow
CCA	Common carotid artery
cGAMP	Cyclic guanosine monophosphate-adenosine monophosphate
cGAS	Cyclic GMP-AMP synthase
CNS	Central nervous system
DAPI	4’,6-Diamidino-2-phenyl-indole
DMEM	Dulbecco’s modified Eagle’s medium
dsDNA	Double-stranded DNA
ECA	External carotid artery
FJC	Fluoro-Jade C
GAPDH	Glyceraldehyde-3-phosphate dehydrogenase
GFAP	Glial fibrillary acidic protein
HIE	Hypoxic-ischemic encephalopathy
I/R	Ischemia–reperfusion
Iba1	Ionized calcium-binding adaptor molecule 1
ICA	Internal carotid artery
i.c.v	Intracerebroventricular injection
iNOS	Inducible nitric oxide synthase
i.p.	Intraperitoneal injection
IRF3	Transcription factor 3
MCAO	Middle cerebral artery occlusion
mNSS	Modified neurological severity score
mtDNA	Mitochondrial DNA
NeuN	Neuronal nuclei
NF-κB	Nuclear factor-κB
NIH	National Institute of Health
NLRP3	Nucleotide-binding oligomerization domain (Nod)-like receptor pyrin domain containing 3
OGD/R	Microglia oxygen-glucose deprivation/reperfusion
PBS	Phosphate-buffered saline
PFA	Paraformaldehyde
qPCR	Quantitative polymerase chain reaction
SAH	Subarachnoid hemorrhage
SD	Standard deviation
si-NC	Scrambled siRNA
siRNA	Short interfering RNA
ssDNA	Signal-stranded DNA
STING	Stimulator of IFN genes
TBI	Traumatic brain injury
TBST	Tris-buffered saline containing Tween-20
TTC	2,3,5-Triphenyltetrazolium chloride
TUNEL	Terminal deoxynucleotidyl transferase-mediated dUTP nick end-labeling
