# Supplementary material for: mtDNA-STING Axis Mediates Microglial Polarization via IRF3/NF-κB Signaling After Ischemic Stroke
Source: Front Immunol. 2022 Apr 5;13:860977. doi: 10.3389/fimmu.2022.860977 (PMC9017276; doi:10.3389/fimmu.2022.860977)
Supplement: Supplementary Figure 1 — Experimental design and animal groups. i.p., intraperitoneal injection; i.c.v, intracerebroventricular injection; MCAO, middle cerebral artery occlusion; RT-PCR, real-time polymerase chain reaction; siRNA, short interfering RNA; STING, Stimulator of IFN genes; TTC, 2,3,5-triphenyl tetrazolium chloride solution; mtDNA, Mitochondrial DNA. [file DataSheet_1.doc]

**mtDNA-STING axis mediates microglial polarization via IRF3/NF-κB signaling after ischemic stroke**

**Lingqi Kong, Wenyu Li, Er Chang, Wuxuan Wang, Nan Shen, Xiang Xu, Xinyue Wang, Yan Zhang, Wen Sun, Wei Hu^*^, Pengfei Xu^*^, Xinfeng Liu^*^**

*** Corresponding author:**

E-mail: [xfliu2@ustc.edu.cn](mailto:xfliu2@ustc.edu.cn); [xupengfei1026@126.com](mailto:xupengfei1026@126.com); [andinghu@ustc.edu.cn](mailto:andinghu@ustc.edu.cn);


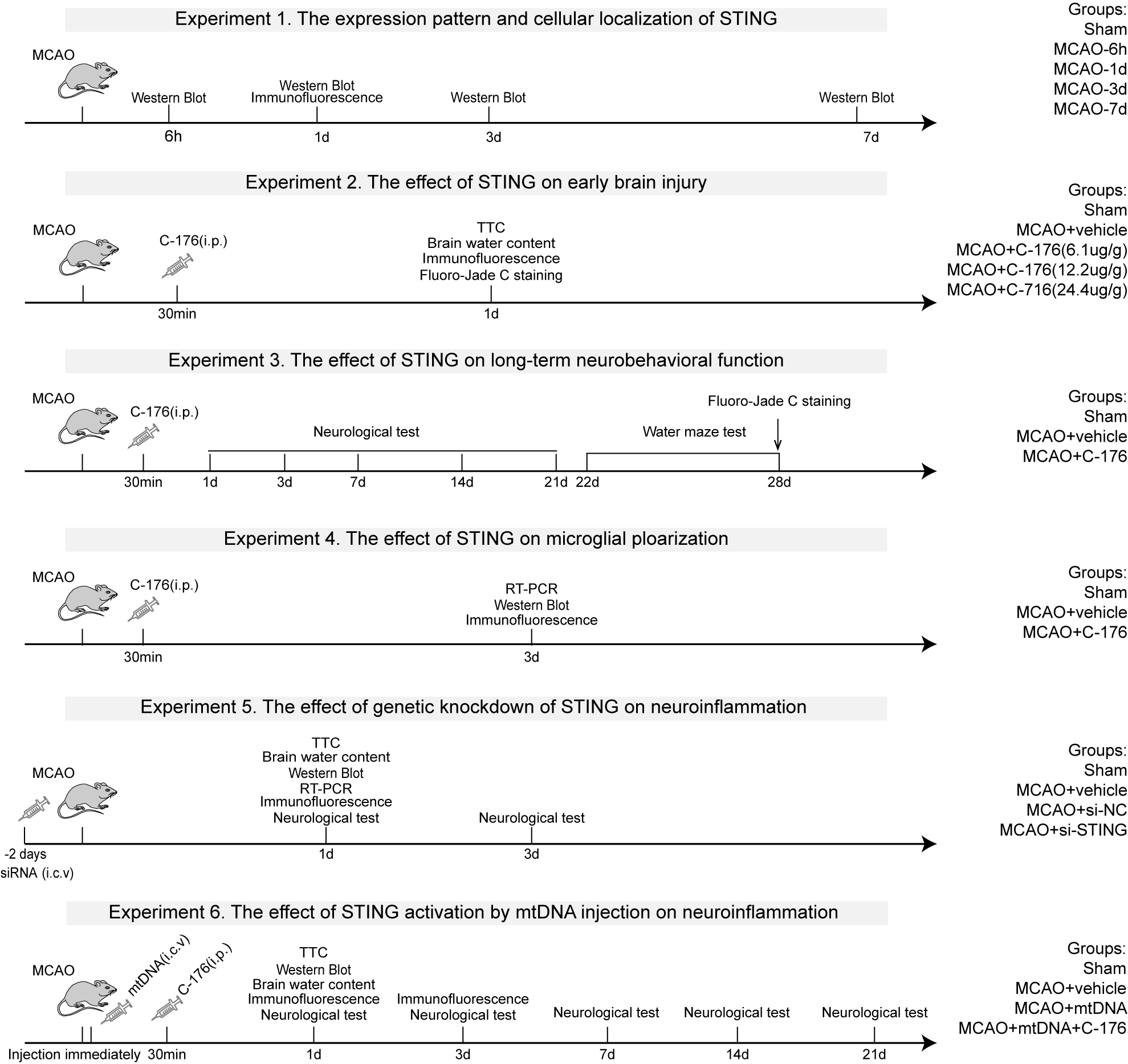


**Fig. S1 Experimental design and animal groups.** i.p., intraperitoneal injection; i.c.v, intracerebroventricular injection; MCAO, middle cerebral artery occlusion; RT-PCR, real-time polymerase chain reaction; siRNA, short interfering RNA; STING, Stimulator of IFN genes; TTC, 2,3,5-triphenyl tetrazolium chloride solution; mtDNA, [Mitochondria](javascript:;)l DNA.

**
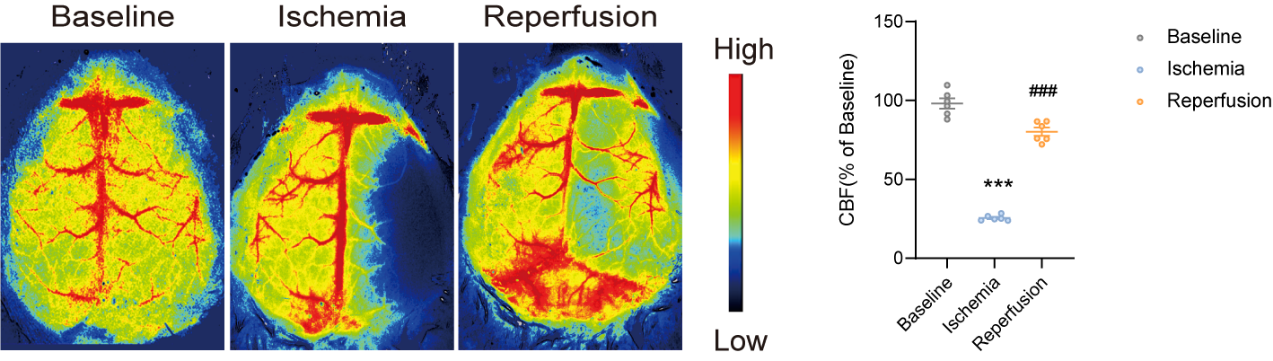
**

**Fig.S2 Occlusion of MCA decreased cerebral blood flow (CBF).** Representative images and quantitative analyses showed the CBF before ischemia, during ischemia, and 24 hours after reperfusion. Data are expressed as mean ± SD, n = 6. ^***^*P* < 0.001 vs Baseline group; ^###^*P* < 0.001 vs Ischemia group.

**
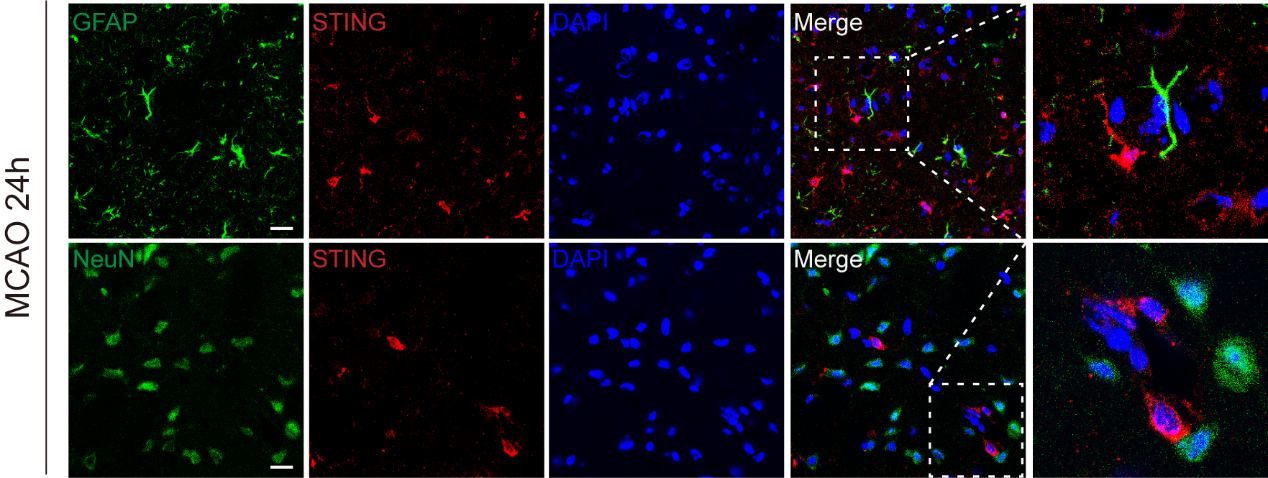
**

**Fig. S3 Colocalization of STING with GFAP or NeuN in the peri-infarct cortex of mice.** STING was undetectable in astrocytes and neurons. Scale bar = 20 μm.


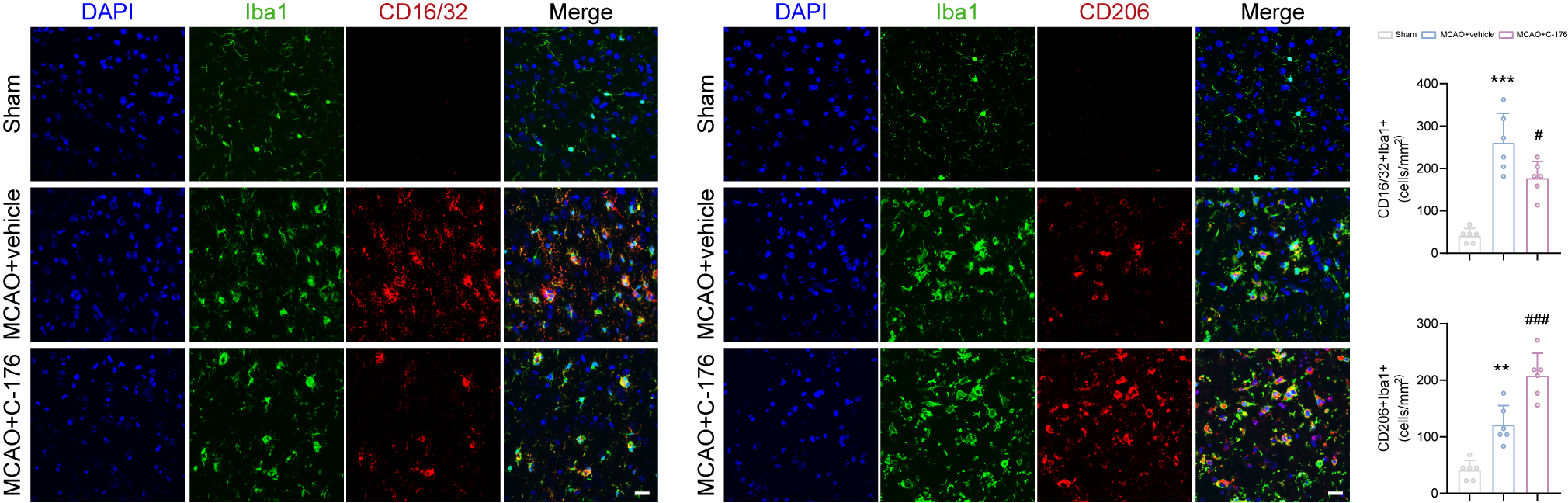


**Fig. S4 Effect of inhibit STING on microglia polarization at 7 days post-MCAO.** Representative images of double immunostaining and quantitative analyses of microglia polarization. M1-phenotype: CD16/32^+^ (red) and Iba1^+^ (green); M2- phenotype: CD206^+^ (red) and Iba1^+^ (green). Data are expressed as mean ± SD, n = 6. ^**^*P* < 0.01, ^***^P < 0.001 vs Sham group; ^#^*P* < 0.05, ^###^*P* < 0.001 vs MCAO+vehicle group. Scale bar = 20 µm.

**
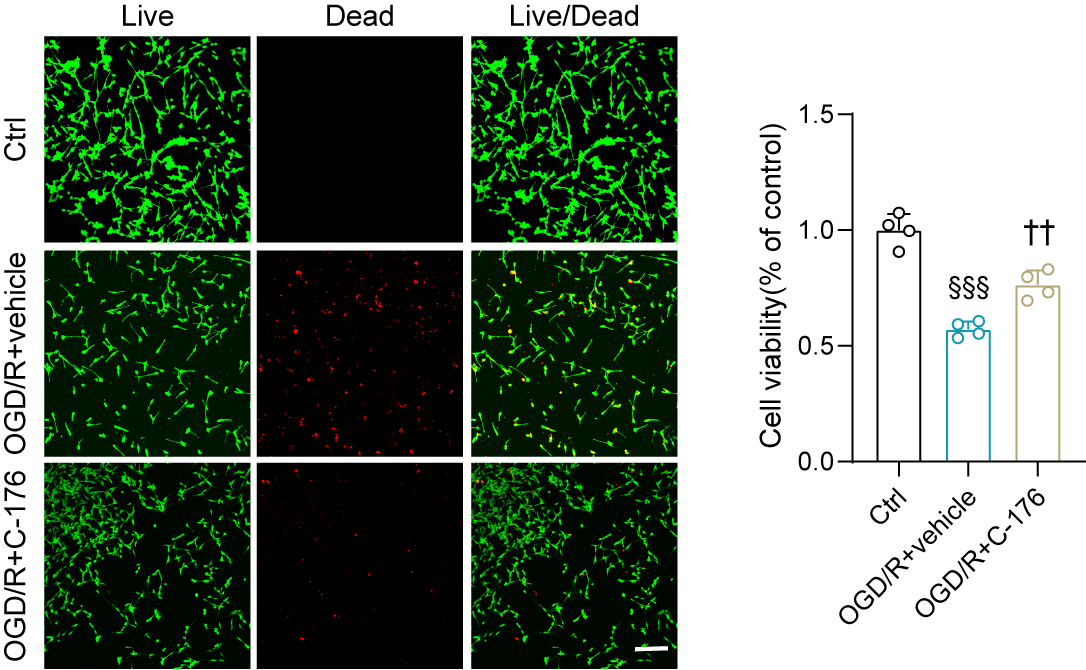
**

**Fig. S5 Pharmacological inhibition of STING suppressed neuronal death in vitro.** Neurons were co-cultured with Control microglia, OGD/R microglia, or OGD/R microglia treated with C-176 (1 μM) for 24 h. Neuronal viability of the three groups was measured by Live/Dead staining. Data are expressed as mean ± SD, n = 4. ^§§§^*P* < 0.001 vs Ctrl group; ^††^*P* < 0.01vs OGD/R+vehicle group. Scale bar = 200 µm.

**
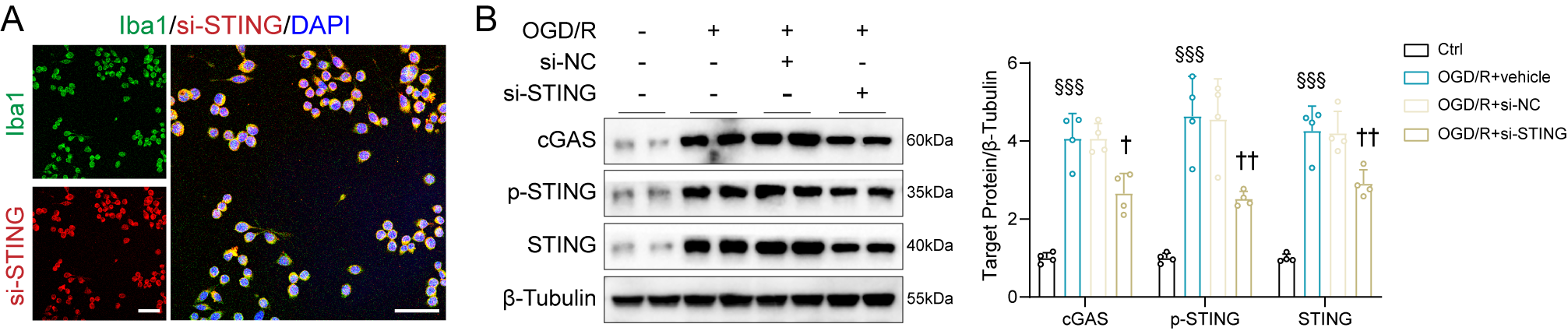
**

**Fig. S6 Transfection of siRNA restrained STING expression in vitro.** (**A**) Colocalization of Cy5-conjugated si-STING with Iba1 24h after OGD. (**B**) Western blotting and quantitative analysis for cGAS, p-STING and STING. Data are expressed as mean ± SD, n = 4. ^§§§^*P* < 0.001 vs Control group; ^†^*P* < 0.05, ^††^*P* < 0.001 vs OGD/R+si-NC group. Scale bar = 50 µm.


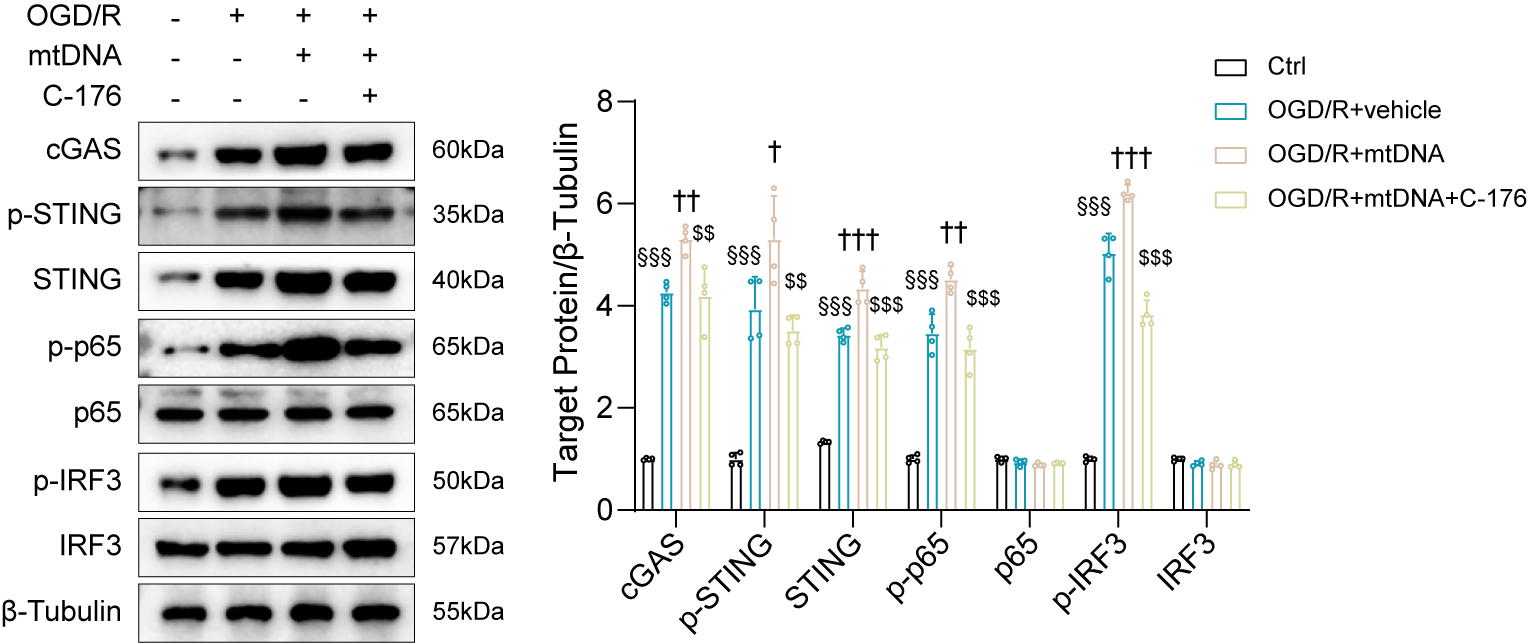


**Fig. S7 mtDNA pre-treatment promoted STING expression in vitro.** BV2 cells were pre-treated with mtDNA (100 ng/ml) for 6h before C-176 incubation. Immunoblots and densitometry analysis of cGAS, p-STING, STING, p-p65, p65, p-IRF3 and IRF3 in Ctrl, OGD/R+vehicle, OGD/R+mtDNA, OGD/R+mtDNA+C-176 groups. Data are expressed as mean ± SD, n = 4. ^§§§^*P* < 0.001 vs Ctrl group; ^†^*P* < 0.05, ^††^*P* < 0.01, ^†††^*P* < 0.001 vs OGD/R+vehicle group; ^$$^*P* < 0.01, ^$$$^*P* < 0.001 vs OGD/R+mtDNA group.

**Supplementary Table S1. Animal usage and mortality of all the experimental groups.**

| **Group** | **Mortality** | **Excluded（reason）** |
| --- | --- | --- |
| **Experiment 1** |  |  |
| Sham | 0% (0/10) | 0 |
| MCAO (6h,1d,3d,7d) | 12.5% (4/32) | 2 (No infarction) |
| **Experiment 2** |  |  |
| Sham | 0% (0/14) | 0 |
| MCAO+vehicle | 18.18% (4/22) | 1 (Cerebral hemorrhage) |
| MCAO+C-176 (6.1ug/g) | 7.69% (1/13) | 0 |
| MCAO+ C-176 (12.2ug/g) | 5.26% (1/19) | 0 |
| MCAO+ C-176 (24.4ug/g) | 0% (0/12) | 1 (No infarction) |
| **Experiment 3** |  |  |
| Sham | 0% (0/15) | 0 |
| MCAO+vehicle | 28.57% (6/21) | 1 (Poor eyesight during water maze test) |
| MCAO+C-176 | 11.76% (2/17) | 0 |
| **Experiment 4** |  |  |
| Sham | 0% (0/6) | 0 |
| MCAO+vehicle | 22.22% (4/18) | 1 (Cerebral hemorrhage) |
| MCAO+C-176 | 12.5% (2/16) | 0 |
| **Experiment 5** |  |  |
| Sham | 0% (0/20) | 0 |
| MCAO+vehicle | 13.04% (3/23) | 0 |
| MCAO+si-NC | 11.11% (3/27) | 1 (Cerebral hemorrhage) |
| MCAO+si-STING | 11.11% (3/27) | 0 |
| **Experiment 6** |  |  |
| Sham | 0% (0/22) | 0 |
| MCAO+vehicle | 18.52% (5/27) | 1 (No infarction) |
| MCAO+mtDNA | 20.83% (10/48) | 2 (Cerebral hemorrhage) |
| MCAO+mtDNA+C-176 | 19.15% (9/47) | 0 |
| **Total** |  |  |
| Sham | 0% (0/87) | 0 |
| MCAO | 15.45% (57/369) | 10 |

**Supplementary Table S2. Antibodies used in this study.**

| **Antibody** | **Manufacturer** | **Catalogue number** | **Dilution** | **Molecular weight (kDa)** |
| --- | --- | --- | --- | --- |
| anti-cGAS | Cell Signaling Technology | #31659 | 1:1000 | 60 |
| anti-p-STING | Affinity Biosciences | AF7416 | 1:2000 | 40 |
| anti-STING | Proteintech | 19851-1-AP | 1:4000 | 35 |
| anti-iNOS | Proteintech | 18985-1-AP | 1:1000 | 130 |
| anti-Arginase-1 | Cell Signaling Technology | #93668 | 1:1000 | 40 |
| anti-β-Tubulin | ABclonal | A12289 | 1:2000 | 55 |
| anti-p-p65 | ABclonal | AP0475 | 1:1000 | 65 |
| anti-p65 | ABclonal | A2547 | 1:1000 | 65 |
| anti-p-IRF3 | Cell Signaling Technology | #29047 | 1:1000 | 50 |
| anti-IRF3 | Cell Signaling Technology | #11904 | 1:1000 | 55 |
| anti-Iba1 | Gene Tex | GT10312 | 1:1000 | - |
| anti-Iba1 | Wako | 011-27991 | 1:1000 | - |
| anti-GFAP | Cell Signaling Technology | #3670 | 1:1000 |  |
| anti-NeuN | Abcam | ab104224 | 1:1000 | - |
| anti-CD16/32 | BD Biosciences | 553142 | 1:500 | - |
| anti-CD206 | R&D Systems | AF2535 | 1:200 | - |
| anti-dsDNA | Abcam | ab27156 | 1:1000 | - |
| anti-HSP60 | Cell Signaling Technology | #12165 | 1:500 | - |
| IgG H&L (Alexa Fluor® 488) | Abcam | ab150105 | 1:1000 | - |
| IgG H&L (Alexa Fluor® 488) | Abcam | ab150073 | 1:1000 | - |
| IgG H&L (Alexa Fluor® 594) | Abcam | ab150132 | 1:1000 | - |
| IgG H&L (Alexa Fluor® 594) | Abcam | ab150068 | 1:1000 | - |
| IgG H&L (Alexa Fluor® 647) | Abcam | ab150107 | 1:1000 | - |
| IgG H&L (Alexa Fluor® 594) | Abcam | ab150156 | 1:1000 | - |

**Supplementary Table S3. Sequences of the PCR primers used in the study.**

| Gene | Primer sequence |
| --- | --- |
| iNOS | FP; AATGCCCGTACCAGGCCCAAT  RP; TAGAGCCCACGCCATCCACTGG |
| IL-1β | FP; TTGTTCATCTCGGAGCCTGTA  RP; CTACTTCCTTTTCTTCCACGA |
| IL-6 | FP; GCACTAGGTTTGCCGAGTAGA  RP; GAGGAAGACACTGAGGTCGAA |
| TNF-α | FP; ATCCGCGACGTGGAACTAG  RP; AAGGTCTTGAGGTCCGCCA |
| IL-10 | FP; GCCTGGGGCATCACTTCTACC  RP; AAGGTCTTGAGGTCCGCCA |
| Arg-1 | FP; TCTTTGGCAGATATGCAGGGA  RP; AAGGTCTTGAGGTCCGCCA |
| UUR | FP; GGCAGAGCCAGGAAATTGC  RP; CACTATTAGGGAGAGGATTTGAACCT |
| COXI | FP; GCCCCAGATATAGCATTCCC  RP; GTTCATCCTGTTCCTGCTCC |
| COX3 | FP; CGTGAAGGAAACTACCCAGG  RP; CGCTCAGAAGAATCCTGCAA |
| ND1 | FP; GGATCCGAGCATCTTATCCA  RP; GGTGGTACTCCCTCTGTAAA |
| GAPDH | FP; AAGAAGGTGGTGAAGCAGG  RP; TGAGGGTGAGAAGGTGGAAG |
